# Single-cell RNA-seq of out-of-thaw mesenchymal stromal cells shows tissue-of-origin differences and inter-donor cell-cycle variations

**DOI:** 10.1186/s13287-021-02627-9

**Published:** 2021-11-04

**Authors:** Camila Medrano-Trochez, Paramita Chatterjee, Pallab Pradhan, Hazel Y. Stevens, Molly E. Ogle, Edward A. Botchwey, Joanne Kurtzberg, Carolyn Yeago, Greg Gibson, Krishnendu Roy

**Affiliations:** 1 School of Biological Sciences, Georgia Institute of Technology, Atlanta, GA 30332 USA; 2 Marcus Center for Therapeutic Cell Characterization and Manufacturing, Institute for Bioengineering and Bioscience, Georgia Institute of Technology, Atlanta, GA 30332 USA; 3 The Wallace H. Coulter Department of Biomedical Engineering, Georgia Institute of Technology and Emory University, Atlanta, GA 30332 USA; 4 Marcus Center for Cellular Cures, Duke University School of Medicine, Durham, NC 27705 USA; 5 Center for Integrative Genomics, Georgia Institute of Technology, EBB 3018, 950 Atlantic Dr NW, Atlanta, GA 30332 USA; 6 School of Medicine, Emory University, Atlanta, GA USA; 7 NSF Engineering Research Center (ERC) for Cell Manufacturing Technologies (CMaT), The Wallace H. Coulter Department of Biomedical Engineering, Georgia Institute of Technology, EBB 3018, 950 Atlantic Dr NW, Atlanta, GA 30332 USA

**Keywords:** MSC, Cell therapy, Bone marrow derived MSC (BM-MSC), Cord tissue derived MSC (CT-MSC), scRNA-seq

## Abstract

**Background:**

Human Mesenchymal stromal cells (hMSCs) from various tissue sources are widely investigated in clinical trials. These MSCs are often administered to patients immediately after thawing the cryopreserved product (out-of-thaw), yet little is known about the single-cell transcriptomic landscape and tissue-specific differences of out-of-thaw human MSCs.

**Methods:**

13 hMSC samples derived from 10 “healthy” donors were used to assess donor variability and tissue-of-origin differences in single-cell gene expression profiles. hMSCs derived and expanded from the bone marrow (BM) or cord tissue (CT) underwent controlled-rate freezing for 24 h. Cells were then transferred to the vapor phase of liquid nitrogen for cryopreservation. hMSCs cryopreserved for at least one week, were characterized immediately after thawing using a droplet-based single-cell RNA sequencing method. Data analysis was performed with SC3 and SEURAT pipelines followed by gene ontology analysis.

**Results:**

scRNA-seq analysis of the hMSCs revealed two major clusters of donor profiles, which differ in immune-signaling, cell surface properties, abundance of cell-cycle related transcripts, and metabolic pathways of interest. Within-sample transcriptomic heterogeneity is low. We identified numerous differentially expressed genes (DEGs) that are associated with various cellular functions, such as cytokine signaling, cell proliferation, cell adhesion, cholesterol/steroid biosynthesis, and regulation of apoptosis. Gene-set enrichment analyses indicated different functional pathways in BM vs. CT hMSCs. In addition, MSC-batches showed significant variations in cell cycle status, suggesting different proliferative vs. immunomodulatory potential. Several potential transcript-markers for tissue source differences were identified for further investigation in future studies. In functional assays, both BM and CT MSCs suppressed macrophage TNFα secretion upon interferon stimulation. However, differences between donors, tissue-of-origin, and cell cycle are evident in both TNF suppression and cytokine secretion.

**Conclusions:**

This study shows that donor differences in hMSC transcriptome are minor relative to the intrinsic differences in tissue-of-origin. hMSCs with different transcriptomic profiles showed potential differences in functional characteristics. These findings contribute to our understanding of tissue origin-based differences in out-of-thaw therapeutic hMSC products and assist in the identification of cells with immune-regulatory or survival potential from a heterogeneous MSC population. Our results form the basis of future studies in correlating single-cell transcriptomic markers with immunomodulatory functions.

**Supplementary Information:**

The online version contains supplementary material available at 10.1186/s13287-021-02627-9.

## Background

Mesenchymal Stromal Cells (MSCs), often referred to as Mesenchymal Stem Cells or Signaling Cells, are cells isolated from various tissues that have shown multipotent, regenerative, and immunomodulatory capacities in vitro. These cells, from a variety of tissue-sources, are being evaluated for therapeutic interventions, especially across a variety of inflammatory and immune conditions [1, 2]. Numerous clinical trials have focused on the use of MSCs as a cell therapy for various diseases with unmet medical challenges, including graft-vs-host disease, osteoarthritis, autism, acute respiratory distress syndrome (ARDS), autoimmune diseases, and even COVID-19. A ClinicalTrial.gov search (date: August 7, 2020) with the keyword: MSC as other terms shows 4,044 studies that are either recruiting, not yet recruiting, enrolling by invitation, and active but not recruiting. MSCs are also widely used in developing engineered tissues ex vivo [3–5]. Several laboratories are also working on developing MSC-based therapies and others are developing reagents and large-scale cell banks for eventual clinical use.

Despite such widespread interest in academia, clinical trials, and in industry, the characteristics of MSCs that are most correlative to their specific in vivo function remain unknown. MSCs can be isolated from various tissues, such as bone marrow, umbilical cord, placental, and adipose tissue, which introduces tissue-dependent variability between MSC-based cell products that may also differ according to donor. Furthermore, manufacturing processes vary between sites (both clinical and commercial), leading to process-dependent variability. These sources of variabilities across the MSC field confound the ability to compare clinical trial results and have contributed to a lack of conclusive historical data to support their potential for clinical use [6].

The International Society for Cell and Gene Therapy (ISCT) standards identify MSCs based on the expression status of a panel of specific surface markers, their ability to adhere to plastic, and their ex vivo tri-lineage differentiation potential to adipocytes, osteoblasts and chondroblasts [7, 8]. However, these minimal MSC identification and functional criteria, especially surface marker expression, often do not correlate with their regenerative or immunomodulatory functions [9]. Moreover, the proportion of “stromal” like progenitor cells that have high regenerative capability varies across MSC donors [10].

Therefore, there is an obvious need for deep phenotypic characterization of MSCs to compare heterogeneity as a function of tissue-of-origin as well as donor, and to identify potential phenotypic signatures that can be eventually used as predictive biomarkers or critical quality attributes (CQAs) for MSC-based products, and for understanding their putative Mechanisms of Action (MoAs).

Recently, single-cell RNA sequencing (scRNA-seq) has emerged as one of the next generation cell characterization techniques that can be used to gain deeper insight into gene transcriptional signatures at the single-cell level [11, 12]. scRNA-seq enables the examination of genomes or transcriptomes of individual cells, providing a high-resolution view of cell-to-cell variation or heterogeneity within a population. Moreover, this technique can be used to explore the distinct biology of individual cells and to understand temporal cellular processes and functions, such as differentiation, proliferation, and immune response potential [13]. scRNA-seq has been previously used to characterize hematopoietic differentiation [14–16] and immune cell subsets [17], including dendritic cells, monocytes [18], and innate lymphoid cells [19]. A handful of reports have also used scRNA-seq to characterize differential gene expression in freshly-prepared MSCs from umbilical cord [20], adipose tissue [8], Wharton’s jelly [21] and bone marrow [22, 23].

In many clinical trial settings for allogeneic MSC-based off-the-shelf cellular therapies, to circumvent logistical and manufacturing challenges, MSC products are used out of thaw (directly from a frozen vial), rather than fresh (without freezing after culture), or culture-rescued (re-cultured after thawing) [2, 24]. Since out-of-thaw MSC products could have different metabolic and functional characteristics from their fresh counterparts [24], phenotypic and functional characterization directly on the out-of-thaw MSC product is necessary to be able to find correlative attributes between their in vitro cell characteristics and corresponding clinical or pre-clinical efficacy. A comprehensive characterization of out-of-thaw MSC product from different tissue sources and donors at single-cell level may provide information on potential critical quality attributes (CQAs) and Mechanisms of Action (MoA) of the cells, and can be used to select MSC donor and/or sources for disease specific cell therapies.

In this study, we performed scRNA-seq using the drop-seq method [11, 25] to compare single-cell transcriptome profiles between commercially available bone marrow-derived MSCs (BM-MSCs) from six donors from RoosterBio Inc. (Frederick MD), and umbilical cord-tissue derived MSCs (UCT-MSCs) from four donors provided by Duke University. We characterized a total of 13 out-of-thaw samples from these ten MSC donors. Specifically, we assessed differences between individual donors as well as differences between MSC tissue sources.

## Methods

### Study approval

This study was approved by the ethics committee of the institutional review boards at Georgia Institute of Technology and Duke University (IRB Protocol No. H17348). All procedures involving human participants were in accordance with the ethical standards of the research committee. Informed consent was obtained from all participants.

### Human bone marrow MSC collection

Seven frozen human bone marrow-derived MSC lots from six male donors were purchased from RoosterBio Inc., and expanded using the RoosterBio expansion protocol (https://www.roosterbio.com/wp-content/uploads/2019/10/A.-RoosterBio-MSC-001-BOM-Expansion-Protocol-IF-08022016.pdf). Briefly, a BM-hMSC high performance media kit was brought to room temperature. Then 1 vial of Media Booster GTX (RoosterBio, catalog no. SU-003) was added to 500 mL hMSC high performance basal media (RoosterBio, catalog no. SU-005). The 10 million BM-hMSC vial was obtained from a liquid nitrogen dewar and immediately thawed at 37 °C for approximately 2 min while monitoring the process and removed from the water bath once a small bit of ice remained. Cells were aseptically transferred to a 15 mL centrifuge tube and 10 mL cultured media was added. The cells were spun down at 200 g for 10 min and all the supernatant was discarded. The cell pellet was re-suspended in 10 mL of culture media and transferred into a 500 mL media bottle. The cells were mixed by capping and gently inverting the bottle and distributed (seeded at 3500–4000 cells/cm^2^ and 42 mL media/T225) equally into T-225 vessels (Corning Cat No. 431082). The vessels were transferred to a 37 °C incubator ensuring that the surfaces were covered with media. The vessels were observed microscopically from day 1 to determine percentage confluency. Once they reached > 80% confluency, they were harvested the next day, and cryo-preserved in Cryostor CS-10 freezing media. All single-cell RNA-sequencing was performed on the out of thaw cells directly from these frozen vials. The surface marker characterization was performed to confirm MSC identification prior to receipt of the cells by RoosterBio Inc.

Samples BM1 and BM2a were cultured in Laboratory A, while samples BM2b, BM3, BM4, BM5 and BM6 were cultured in Laboratory B. Samples BM2a and BM2b were from the same donor.

### Human cord tissue MSC collection

For cord tissue derived samples, six frozen human MSC samples from four male donors were provided by the Department of Pediatrics, Duke University. Cryopreserved P0 vials were placed in a sterile bag which was itself placed in a 37 °C water bath. Vials were thawed until the cell suspension was slushy (~ 2 min). Cell suspensions from the vials were transferred to a 15 mL tube containing XSFM (Irvine Scientific, cat. no. 91149) using a sterile serological pipette and the cell suspension was mixed slowly. The cryovial was rinsed with 0.5 mL of XSFM and the rinse was transferred to the 15 mL tube. After mixing slowly, the cell count and viability was measured. The cells were mixed in the 15 mL conical using a sterile pipette and the volume containing 3.4 × 10^6^ cells was transferred into a bottle containing 1.12 L of XSFM. The bottle was mixed gently and then poured into HYPER flasks. The HYPER Flasks were placed into a 37 °C/5% CO2 incubator. The P1 cells were harvested after 5–7 days. The P2 cells were then frozen using CS-10 freezing media and cryopreserved. The Cryopreserved P2 cells were shipped to us for the downstream characterizations. The surface marker characterization was performed to confirm MSC identification prior to receipt of the cells by Duke University. All the single-cell RNA-sequencing was performed on the out of thaw cells directly from these frozen vials.

Samples UCT1a, UCT1b and UCT1c come from the same donor.

### Thawing and single cell suspension preparation for single-cell RNA-sequencing

Frozen vials containing 1 million MSC were thawed in a 37 °C water bath for a couple of minutes. Cells were then aseptically transferred to a 15 ml centrifuge tube. Room temperature RPMI media (1 mL) was used to rinse the cell vial and added to the cells in the 15 ml tube. Another 3 ml of media was added to the cells and mixed well with a serological pipette. Cells were counted using a NucleoCounter (Chemometec) and spun down at 200 g for 10 min. The cells were re-suspended in media and counted again and processed for scRNA-sequencing.

### Single-cell RNA-seq library preparation and sequencing

The Illumina-Bio-Rad ddSEQ platform was used to process, capture, and barcode the cells to generate single-cell Gel Beads by following the manufacturer’s protocol. Cell suspensions were loaded onto a ddSEQ Cartridge along with reverse transcription master mix, and encapsulated and barcoded by the Single-Cell Isolator. Lysis and barcoding took place in each droplet. Droplets were disrupted and cDNA was pooled for second strand synthesis. Libraries were generated with direct cDNA tagmentation using Nextera technology. Tagmentation was followed by 3′ enrichment and sample indexing to prepare indexed, sequencing-ready libraries. The libraries were sequenced using Nextseq sequencing Platform on an Ilumina NextSeq in the IBB Molecular Evolution core at Georgia Tech (PE75, mid-output V2.5 kit). The library quality (check for primer dimers, adopter dimers, ethanol contamination, degradation as well as the size and concentration) was confirmed before each sequencing run using the Agilent Bioanalyzer2100.

All the BM-MSC samples have 2415 cells with an average 3,835 genes/cell (Table 1) and the UCT-MSCs have 1785 cells with average 3056 genes/cell (Table 2). The BM-MSC samples have an average 28,890,505 (Table 1) reads per sample and the UCT-MSC samples have 33,771,805 (Table 2) reads per sample on average.

**Table 1:** Number of cells, average number of genes and average number of UMI per cell per BM-MSC sample

Samples	Lab	Number of cells	Number of reads	Mean reads per cell	Median genes per cell	Number of cells post filtering	Sex	Age-range	Average nGenes per cell	Average nUMI per cell
BM1	A	293	29,671,530	15,469	2218	293	Sex-A	21–30	2693	7581
BM2a	A	310	29,943,109	46,467	3855	252	Sex-A	21–30	3714	12,242
BM2b	B	199	41,511,093	39,417	4686	199	Sex-A	21–30	4395	15,506
BM3	B	590	35,842,779	26,580	4272	452	Sex-A	21–30	4193	13,415
BM4	B	557	25,262,984	17,668	3803	542	Sex-B	21–30	3430	9718
BM5	B	149	18,160,926	32,928	4201	122	Sex-A	31–45	4057	13,591
BM6	B	317	21,841,113	29,393	4554	275	Sex-A	18–30	4367	14,385

**Table 2:** Number of cells, average number of genes and average number of UMI per cell per UCT-MSC sample

Samples	Lab	Number of cells	Number of reads	Mean reads per cell	Median genes per cell	Number of cells post filtering	Sex	Age-range	Average nGenes per cell	Average nUMI per cell
UCT1a	C	161	16,255,153	19,563	2472	161	Sex-A	–	2808	8458
UCT1b	C	251	31,333,645	38,809	3840	251	Sex-A	–	3667	12,075
UCT1c	C	251	31,333,645	38,809	3840	251	Sex-A	–	2477	7321
UCT2	C	692	59,783,150	25,248	2979	547	Sex-A	–	3053	9381
UCT3	C	397	43,757,708	26,241	2724	396	Sex-A	–	2720	9262
UCT4	C	284	20,167,531	29,162	3717	259	Sex-A	–	3616	11,711

Confirmation that MSCs were relatively pure populations of undifferentiated cells was revealed by FACS analysis of cell surface markers provided by the manufacturer as release criteria. Furthermore, scRNA-seq (which is less sensitive due to high drop-out rates) confirmed prevalent expression of *NT5E* (CD73), *THY1* (CD90), and *ENG* (CD105) and absent expression of *CD34* among other genes (Fig. 6). *ENG* was expressed on 66% of the cell, *NT5E* on 72%, and *THY1* on 92%. In contrast, each of the transcripts *PTPRC, CD34, CD14, ITGAM, CD79A, CD19*, and *HLA-DRA* were detected in less than 0.5% of the cells.

### Data analysis

Sample demultiplexing and gene counts were extracted using the Illumina Sure cell pipeline. The raw reads were trimmed, and the gene-barcode matrix was generated. Surecell was also used to filter and align the samples and to generate gene-cell UMI count matrices. The seven samples from bone marrow were sequenced in three different batches, and the six samples from cord tissue were sequenced in two batches.

Cells with mitochondrial expression greater than 5% or low gene counts were removed, as were genes expressed in fewer than one percent of the cells. Downstream analysis was initiated with SC3 software [26] for cell clustering. Cells were normalized to counts per 10,000 before using Seurat [27] for differential expression estimation. Wilcoxon rank sum test was performed in Seurat [27], negative-log P-values were computed, and genes significant with an adjusted P-value less than 5% were selected for downstream gene ontology analysis. Then gene ontology was performed on the differentially expressed genes using GSEA [28, 29] and ToppGene [30] tools.

**Fig. 1:**
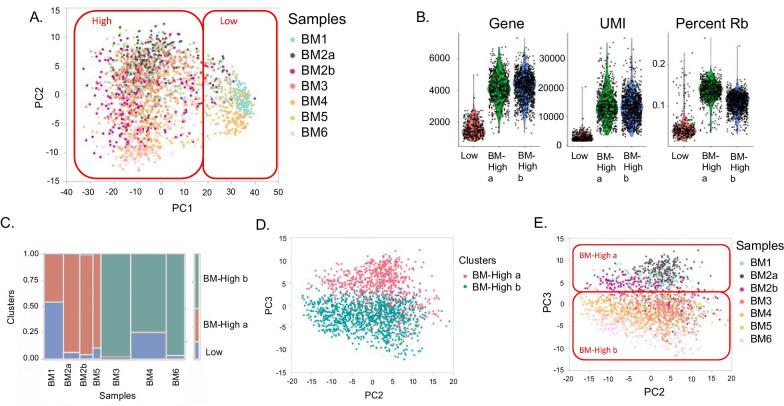
Clusters of BM-MSC transcriptome profiles. **A** The first two Principal Components of gene expression identify two broad clusters of cells, which are colored by sample: Cluster BM-Low, which corresponds to low UMI count cells and cluster BM-High, with high UMI count cells. **B** Violin plots show the density of the number of Genes, UMI, and Ribosomal Protein transcripts (RP) per cell. **C** Association of cells with clusters. The width of each column is proportional to the number of cells in the indicated [47] sample, and the color of each box corresponds to cells in cluster BM-Low (blue), BM-High_a (red) or BM-High_b (green). **D** Within cluster High, SC3 identifies two clusters of cells, which separate along PC3 as indicated by the red and blue points. **E** Shading of cells by sample confirms that cells from each donor belong to one of the two sub-clusters, although with subtle separation associated with PC3

Two technical replicates for the samples RB179 and RB139 were used to analyze the variance due to batch effects. These cells were expanded and frozen in aliquots on the same day of harvest. The replicates were thawed from the frozen aliquots on different days and processed for single cell RNA-seq on the Bio-Rad ddSeq platform. Principal variance component analysis on the 4 samples assessing the weighted contributions of effects to the first 10 PC, showed that the major variance source is the three cell clusters (60.1% of the total variance), followed by donor and batch related variability at 6.6% and 4.1% respectively. Interaction effects were negligible. This analysis shows that some of the differences among donors are actually attributable to thaw effects, though we emphasize that in both the BM and CT datasets, independent thaws clustered together (Figs. 1E, 3C).

## Results

### Donor effects on bone marrow-derived MSC gene expression

A total of seven bone marrow-derived MSC (BM-MSC) samples from six donors were thawed and processed for scRNA-seq analysis (Tables 1, 2). First, we focused our analyses on post-freeze MSC products from BM and CT origins, since these are being widely used in numerous clinical trials.

Table 1 summarizes the sample data for all BM samples. Samples BM1 and BM2a were processed in laboratory A, while samples BM2b, BM3, BM4, BM5 and BM6 were processed in laboratory B (Table 1). An average of 305 cells were profiled per sample, with an average read depth of 29,703, representing 12,348 UMI (Unique Molecular Identifier) and 3836 expressed genes per cell (Table 1). The profiles were clustered with both Seurat [27] and SC3 pipelines [26]. Since the latter is optimized for relatively small experimental designs, we present the results of SC3, but note that similar findings were obtained with Seurat (Additional file 1: Fig. S2). Before characterizing differential expression among samples, we confirmed that the MSC-identity markers established by the ISCT, namely *NT5E (*CD73), *THY1* (CD90) and *ENG* (CD105) were detected in the majority of cells in the bone marrow and umbilical cord tissue derived MSCs (Additional file 1: Fig. S3). Furthermore, *CD34, CD14, CD19* and *PECAM1*—all markers of hematopoietic or lymphoid lineages, were absent.

Projecting each cell against the first two Principal Components (PC) of gene expression, three clusters of single cell profiles were observed (Fig. 1A). PC1 is highly negatively correlated with the total UMI count per cell. Accordingly, the smallest cluster located to the right, consists of 17% of the cells, all of which had low UMI counts, typically fewer than 1000 detected genes, and low ribosomal protein transcript counts (Fig. 1B). These low UMI counts' cells were more prevalent in two donors studied—one from each of the two laboratories (Lab A and Lab B; BM1 and BM4, respectively: Fig. 1C), suggesting the low UMI count may not be related to the lab in which they were manufactured. It is not clear whether the unusual profile of these cells is a technical artefact, or has a biological basis, but they appear to be of low quality and were excluded from all subsequent analyses.

Two clusters identified by SC3 in the remaining high-quality datasets largely differentiate along PC3 (Fig. 1D). Three of the five samples expanded in laboratory B (BM3, BM4 and BM6) were predominantly found in cluster BM-High_b; the other two samples along with both of the samples expanded in laboratory A (BM1, BM2a, BM2b and BM5) were predominantly found in cluster BM-High_a (Fig. 1E). Both samples from the donor whose cells were cultured in each of the laboratories are in cluster BM-High a (BM2a and BM2b), suggesting that the difference is more likely to be donor-related than due to a laboratory or technical effect. Nevertheless, Fig. 1E shows that even between the two laboratories, the cells from this donor tend to separate along PC3.

### Single cell differential expression analysis for bone marrow-MSC samples

**Fig. 2:**
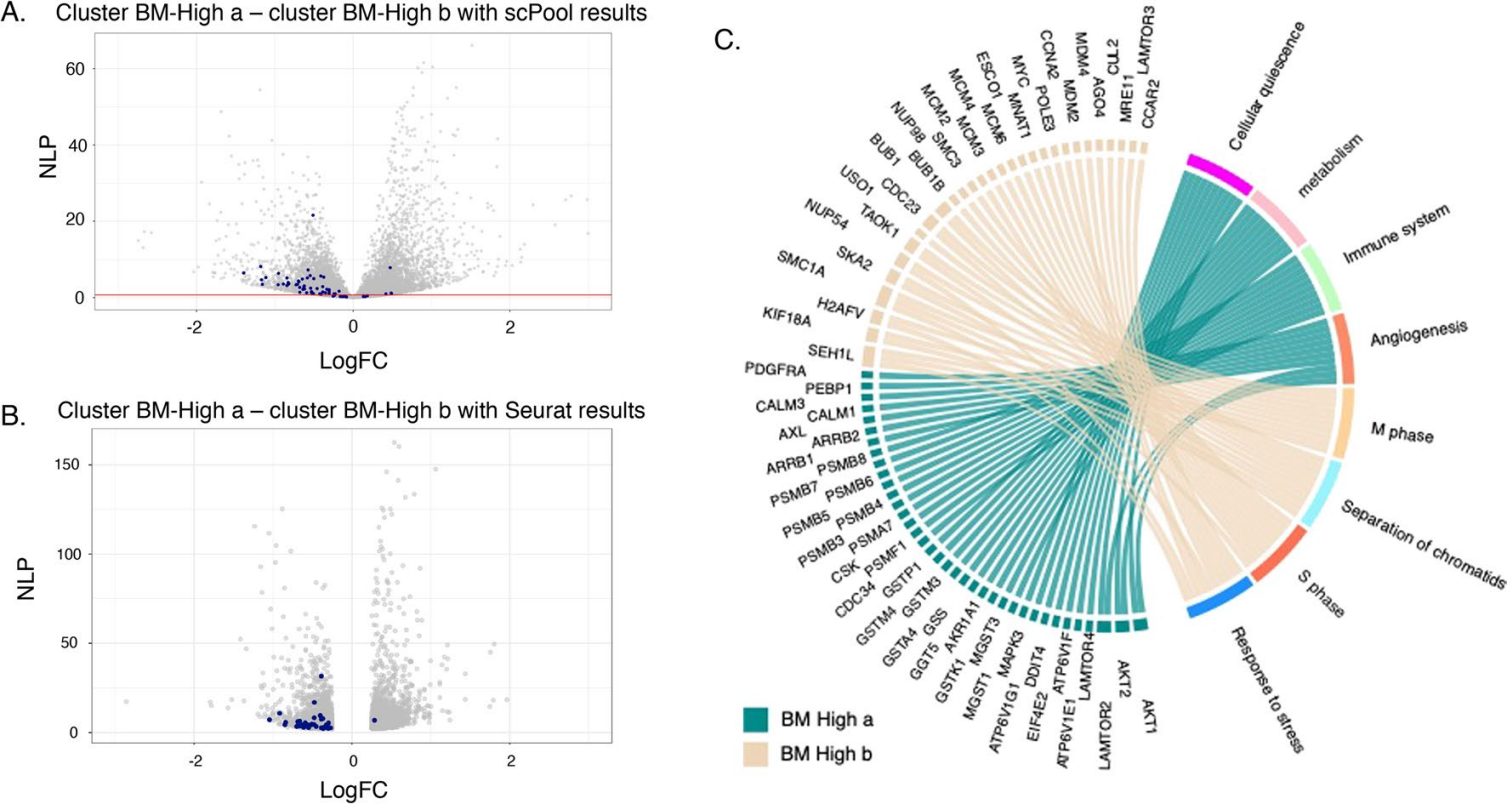
Differential expression between the two High clusters of Bone Marrow-MSC samples. **A** Volcano plot of negative log *P* value (NLP) against fold change, created with standard single cell level computation in  scPool and Seurat. **C** Chord diagram of gene ontology analysis highlighting the top 10 differentially expressed genes in each of 8 pathways representative of the up- and down-regulated genes. Ribbons link genes on the left to pathways on the right; genes associated with multiple pathways bifurcate. Note that in this depiction, the direction of differential expression is the same for all genes in the pathway

After normalizing gene expression values to counts per 10,000 UMI, differential expression analysis between clusters BM-High_a and BM- High_b was performed in Seurat using the Wilcoxon rank sum test, yielding 1667 genes at a adjusted *P* value of 5%. There were 767 genes upregulated in cluster 2a, and 900 upregulated in cluster 2b (Fig. 2A). Gene ontology analysis detected strong enrichment for multiple pathways involved in cell cycle regulation in cluster BM-High_b (Fig. 2B). By contrast, cluster BM-High_a showed upregulation of multiple pathways related to immune signaling and other processes expected to be characteristic of functional MSCs (Fig. 2B). On the basis of the cell cycle gene expression, cells in cluster BM-High_b may be preparing for, or undergoing cell cycle division, whereas the cluster BM-High_a MSCs are more likely to be in G0 phase. Alternately the two populations may simply express cell cycle related genes at different levels, without this reflecting cell cycle stages.

Next, we probed the magnitude of donor contributions to variance within clusters, by performing analysis of variance with donor as the fixed effect of interest. Within just the high-quality cluster BM-High_a cells, donor effects accounted for 8.5% of the variance. A similar result was observed for cluster BM-High_b.

### Donor effects on umbilical cord tissue derived MSC gene expression

Umbilical cord tissue derived MSC (UCT-MSC) samples from four donors were used for scRNA-seq analyses. A total of six scRNA-seq samples were prepared, all from the same lab, including three biological replicates of one donor sample (UCT1a, UCT1b and UCT1c). An average of 349 cells were profiled per sample, with an average read depth of 27,417, representing 9700 UMI and 3057 expressed genes per cell (Table 2). The UCT-MSC gene expression profiles were also analyzed with the SC3 pipeline.

**Fig. 3:**
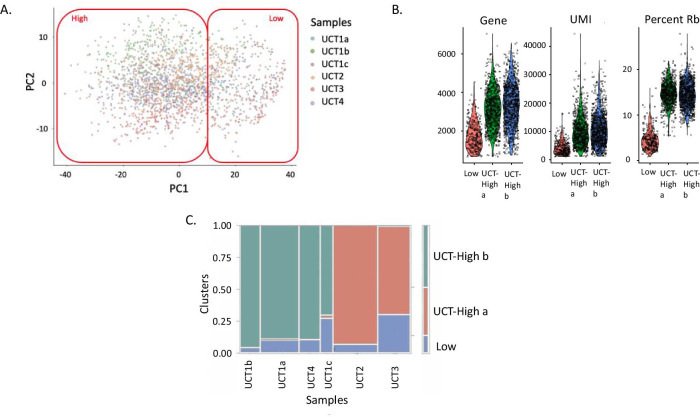
Clusters of UCT-MSC Profiles. **A** The first two Principal Components of gene expression identify the two major clusters of cells, which are colored by sample. Most cells of each sample cluster together. **B** Violin plots show the density of the number of Genes, UMI, and Ribosomal Protein transcripts (RP) per cell. **C** Association of cells with clusters. The width of each column is proportional to the number of cells in the indicated sample, and the color of each box corresponds to cells in cluster 1 (blue), 2a (red) or 2b (green)

Two major clusters of single cell profiles were again observed in the projection of the first two principal components of the UCT-MSC data (Fig. 3A). The smallest of these, consisting of 13% of the cells, was characterized by cells with low UMI counts, typically fewer than 2,000 detected genes (Fig. 3B), similar to the BM-MSC analysis. These low UMI-count cells were present in every sample but again were more prevalent in two of the samples (UCT1c and UCT3: Fig. 3C). It is not clear whether the origin of these cells is a technical artefact, or has a biological basis, but they also appear to be of low quality and were again excluded from all subsequent analyses. Within the high-quality cells, SC3 once more identified two clusters of cells, UCT-High_a (UCT2 and UCT3) and UCT-High_b (UCT1a, UCT1b, UCT1c and UCT4), though in this case they did not clearly correspond to one of the Principal Components. The three replicates of donor UCT1 were primarily captured within the UCT-High_b cluster, suggesting consistency of technique.

**Fig. 4:**
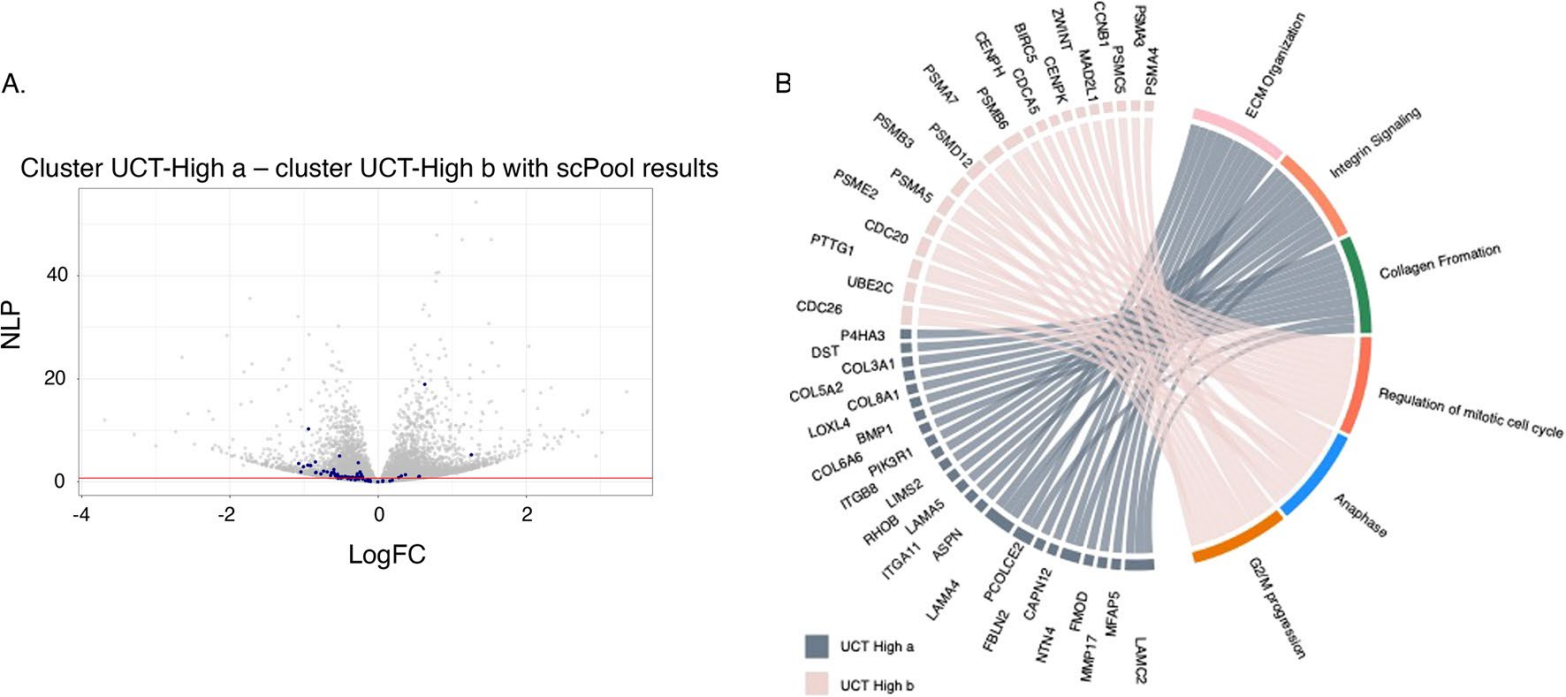
Differential expression between two high quality UCT-MSC clusters. **A** Volcano plot of significance against fold difference in gene expression for the comparison of clusters 2a and 2b. Blue points indicate genes with established roles in cell-cycle regulation. **B** Chord diagram of gene ontology analysis highlighting the top 10 differentially expressed genes in each of 10 pathways, representative of the up- and down-regulated genes. Ribbons link genes on the left to pathways on the right; genes associated with multiple pathways bifurcate

Implementation of the Wilcoxon rank sum test for detecting differential gene expression between the two UCT-MSC clusters, after removing the low-quality cells, detected 587 genes at an adj P-value of 5%. Directional up-regulation of established marker genes for mitosis is evident in cluster UCT-High_b as indicated by blue points in the volcano plot Fig. 4A. Gene ontology analysis (Fig. 4B) indicates enrichment for up-regulation of collagen biosynthesis, integrin signaling, extracellular matrix (ECM) organization, and protein translation pathways in the cluster UCT-High_a MSCs, whereas the cluster UCT-High_b cells are enriched for cell cycle regulation, degradation of mitotic proteins, as well as various processes related to cell cycle progression, including *CDC20* mediated degradation of Securin, and auto degradation of *CDH1*, suggesting potential donor-dependent heterogeneity in the gene expression profile of UCT-MSC.

### Comparison between bone-marrow and cord-tissue derived MSC single-cell gene expression profiles

**Fig. 5:**
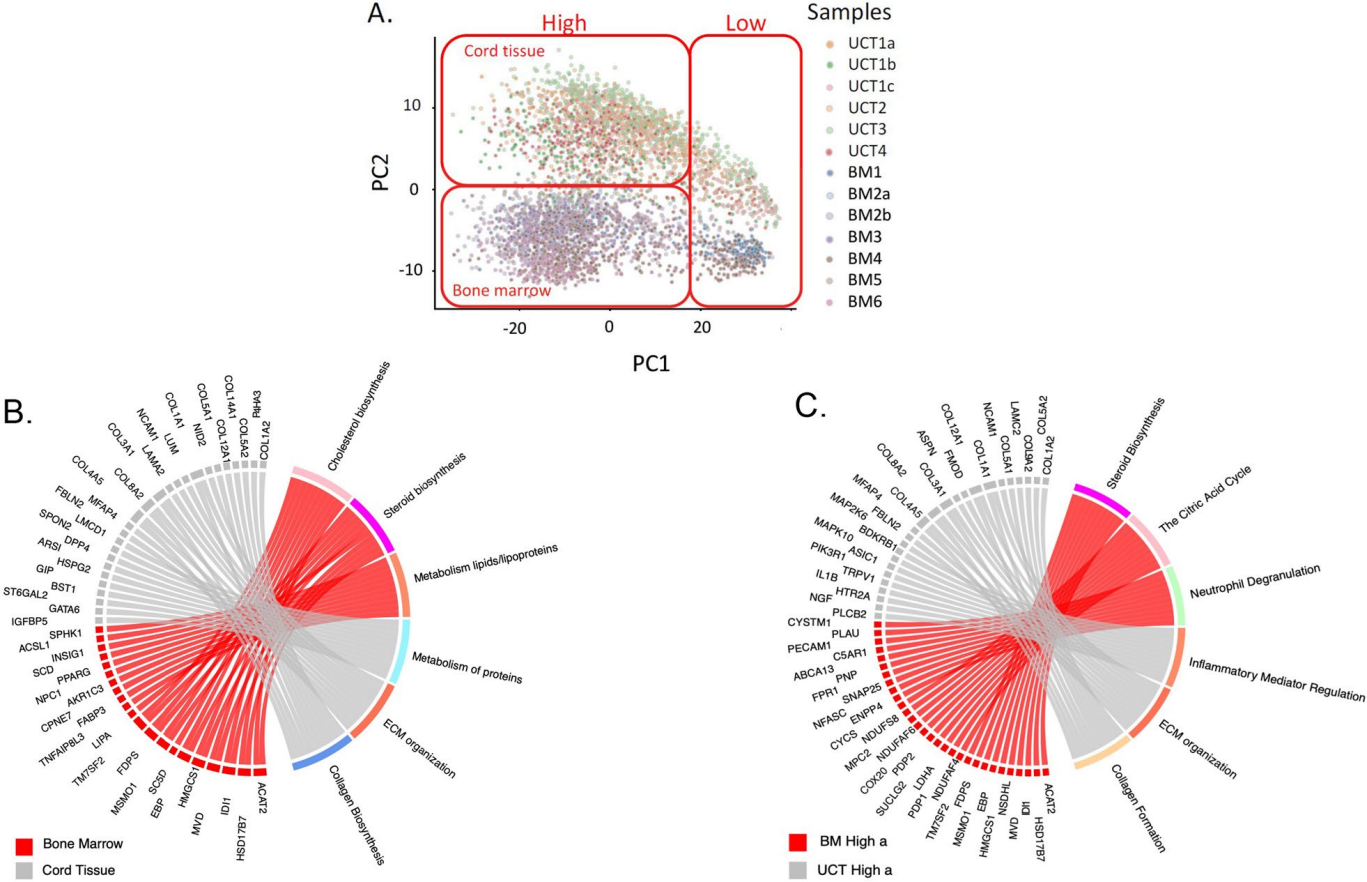
**A** Principal component analysis showing clustering of MSC samples by tissue of origin (bone marrow vs umbilical cord tissue). **B** Chord diagram summarizing differential expression between UCT-MSC and BM-MSC. **C** Cluster-specific pathway expression is not conserved between the two High clusters from the two tissues of MSC origin. Chord diagram contrasting clusters BM-High_a versus UCT-High_a

Direct comparison of MSCs derived from bone marrow and MSCs from umbilical cord tissue was performed by combining the analyses of the previous two datasets. As expected, Principal Component Analysis (PCA) of the raw single cell profiles again identified two major clusters of low and high UMI-abundance cells along PC1, but in this case PC2 of the joint analysis cleanly differentiates the BM and CT samples (Fig. 5A). This result implies that there are significant differences in gene profile between MSCs derived from the two source tissues.

2612 genes were found to be up-regulated in the BM-MSC, and 689 genes down-regulated, compared to UCT-MSCs. We highlighted the top 16 differentially expressed genes (Additional file 1: Fig. S4). Gene ontology analysis of all the significantly differential expressed genes indicates enrichment for metabolism of lipids and lipoproteins, cholesterol biosynthesis, mitochondrial translation, and metabolic pathways in the BM-MSCs, whereas the UCT- MSCs were enriched for ECM organization, collagen biosynthesis and signal transduction (Fig. 5B). UCT-MSC also showed relative up-regulation of mitotic cell cycle pathways, but this likely reflects the greater ratio of UCT-High_b to UCT-High_a cluster cells than of BM-High_b to BM-High_a cells, rather than a consistent trend favoring cell division in the UCT-MSC.

Pathway enrichment analysis also showed that the differences between the two clusters in each tissue are not consistently maintained. The chord diagram (Fig. 5C) highlights pathways overexpressed in BM-High_a and UCT-High_a, which are not the same. These data confirm differences in the gene expression of non-dividing cells as a function of tissue of origin and suggest that the two types of MSCs are likely to have divergent regulatory and functional potentials. Importantly, the UCT-High_a population exhibit higher expression of genes involving pro inflammatory mediation, ECM organization and collagen biosynthesis, whereas the BM-High_a population had higher expression of steroid biosynthesis, the citric acid cycle and neutrophil degranulation genes (Fig. 5C).

Next, we examined the expression level of genes that play important roles in the immunomodulatory response induced by MSCs. Focused comparison of expression of genes that are associated with cell adhesion, migration, immunosuppression and immunostimulation between the BM- and UCT-derived MSCs suggests tissue-of-origin and donor differences in gene activity (Additional file 1: Fig. S5). BM-High_a cells characteristically overexpress transcripts encoding the membrane proteins prostaglandin synthase (*PTGES2*) and Endoglin (*ENG*) as well as the lysosomal protein CD63, compared to BM-High_b, whereas BM-High_b cells overexpress the genes *CD46* (encoding a complement cofactor), *CD47* (an integrin-associated protein), and *CD146* (*MCAM*, cell adhesion molecule), compared to BM-High_a. These genes are in general overexpressed in BM derived MSC compare to UCT derived MSC. BM derived MSCs have higher expression of the cell surface glycoprotein coding genes *CD44* and *CD59*, as well as the nucleotidase *NT5E* and immune checkpoint molecule *CD276* compared to UCT derived MSC. Conversely, when comparing UCT-High_a and UCT-High_b clusters, UCT-High_a cells overexpress the tetraspanin regulators of motility *CD151*, and cell surface protein coding genes *CD99*, *THY1* and *CD248*, while UCT-High_b overexpressed *CD9*. The cell surface protein coding gene *CD81* is not significantly differentially expressed between the clusters UCT-High_a and UCT-High_b. We looked at two genes associated with immunostimulation: *CCL2* and *CD109*, which are overexpressed in UCT and BM derived MSCs, respectively. Post thaw BM-MSC or CT-MSC, co-cultured with THP-1-derived macrophages, (BioRxiv Pradhan, BM1 and BM6 and CT1-3 (hyperflask-expanded)), downregulated macrophage-mediated TNF alpha production. We also evaluated multiple pluripotent and stemness genes (Additional file 1: Fig. S6), none of which were found to be significantly differentially expressed between bone marrow and umbilical cord tissue.

We briefly compared two of the BM-MSC samples between pre-freeze and post-freeze conditions to understand freeze–thaw effects on MSC gene expression. Our analysis indicated a shift in the genetic profile between pre-freeze and post-freeze conditions (Additional file 1: Fig. S1). Pre-freeze samples showed significant overexpression of 1,743 genes relative to post-freeze samples, at the 5% false discovery rate (FDR) threshold, while 310 genes were significantly overexpressed in the post-freeze samples compared to the pre-freeze samples. Some of the pathways overexpressed in the pre-freeze samples are cytokine signaling (*FOS, MMP2, TLN1, FOSB*), cell proliferation and cell adhesion (*ZYX, ITGA5, CLIC1* etc.), while the pathways over-expressed in the frozen, post-freeze samples are carbohydrate interconversions (*UGP2*), cholesterol/steroid biosynthesis and regulation of apoptosis (*PSMA2, PSMB1*). We acknowledge that to understand the impact of these differences we need further extensive study with more data and statistical power along with validation.

## Discussion

MSCs from various tissue sources are the subject of 4044 registered clinical trials (ClinicalTrials.gov- keyword: MSC as other terms with filters—not yet recruiting, recruiting, enrolling by invitations, and active, not recruiting; search date—August 7, 2020). It is thus important to develop robust high-throughput approaches for characterization of diverse batches from various tissue sources in order to help evaluate reasons for success or failure of individual trials or patient responses. Single cell RNA-sequencing is a relatively unbiased approach to profile the molecular attributes of individual cells. The potential utility of scRNA-seq includes characterization of heterogeneity that cannot be observed with bulk RNA-seq, and monitoring of the effect of the stage of the cell-cycle on transcriptional diversity.

Our study is the first to characterize the cryopreserved MSCs from different donors and different tissue sources at the same time at the high throughput single cell level. Here we describe a droplet-based scRNA-seq comparison of donor, tissue-of-origin, and expansion conditions of out-of-thaw MSC variability, concluding that bone marrow and umbilical cord tissue-derived MSCs have significant differential expression that likely explains some of the documented differences between them, and that donor differences are modest yet significant. To our knowledge, six other scRNA-seq studies [11, 12, 20–23] of MSCs have been published, and our results are broadly concordant, though with some important differences in emphasis. Barrett *et al.,* 2019 [22] used a version of SmartSeq to deeply profile 103 Wharton’s jelly-derived umbilical cord MSCs and 63 bone marrow-derived MSCs, identifying 463 differentially expressed genes enriched for activity in numerous processes including the matrisome, coagulation, angiogenesis, and wound-healing via immune-regulation. The current study similarly finds a difference between cord tissue and bone marrow-derived MSCs. Additionally, our data also shows a cell cycle variability which seems to be related to the donor [22].

Each of the other studies [11, 12, 20–23] has noted that the cell cycle gene expression is a major source of heterogeneity within donors. Also, it is important to note all these studies have been done with cells derived from the cultured MSCs and not a frozen product. We emphasize the novelty of our study by characterizing the transcriptome profiles from 2415 BM-MSC cells and 2036 CT-MSC cells with a read depth of up to 46,467 reads per cell (Tables 1, 2). In this study we show the cell cycle related gene expression being the major driving factor of the heterogeneity within donors from our variant component analysis.

According to the *Huang et al.*, 2019 [20] study, it appears the cell cycle is related to the immune regulatory potency of the MSCs. Previous work [31] used a core set of G1/G2M/S markers to assign cells to each phase, and regressed out this source of variance before performing downstream analysis. We eschewed this approach both because of concerns over the reliability of the assignments, and to emphasize that the proportion of cells with low expression of these genes is an important component of among-donor differences in both BM-MSC and UCT-MSC. Reported higher proliferative capacity of Wharton’s jelly-derived MSCs [22] is consistent with the higher proportion of mitotic genes in our UCT-MSCs relative to BM-MSCs. However, it should be emphasized that higher overall expression may not correlate with higher rates of proliferation, since expression levels may vary among donors without implying that a different fraction of cells are undergoing division. On the other hand, it appears that putative G0 cells that do not express cell cycle genes have quite different transcriptional properties that are directly relevant to their biological functions, such as immunomodulatory potential. We note that each of our samples was profiled at population doubling level (PDL) ranged from 12 to 15, eliminating passage number as a source of variability in our study.

Other authors have also chosen to regress out “batch” effects before searching for heterogeneity, even though in each case “batch” appears to be coincident with “donor” [8, 21] or “Passage” [20]. In the absence of biological replication, that is, two MSC preparations obtained independently from the same donor, it is impossible to know whether differences between sample populations have a biological or technical basis. Nevertheless, we estimate from principal component variance analysis that less than 10% of the overall expression variability is among donors/batches within each of the two clusters observed in both the BM- and UCT-MSC datasets (Figs. 1C, 3C). We see this minor source of variability is donor-related in the two instances where we had technical replicates from the same donor (in the case of the two BM-MSC samples cultured in different laboratories). The cells tended to be assigned to the same sub-cluster BM-High_a or UCT-High_a. Whether or not these differences impact MSC function in clinical applications remains to be seen, additional large-scale comparisons with a large set of samples with high quality data on patient outcomes will need to be analyzed. We acknowledge that the use of BM-MSC cultured in two different laboratories is a limitation of the study.

In this study the transcriptomes of human bone marrow and cord tissue-derived MSCs were analyzed via drop-seq single cell RNA-seq. Using this approach, new information about MSCs emerges. First, the differences between bone marrow-derived MSCs and cord-tissue derived MSCs were seen. Surprisingly, pathways up-regulated in G0 bone marrow-derived MSCs did not correspond to the same pathways upregulated in G0 cord tissue-derived MSC (Fig. 5C). Further, we observed differences in various immune regulatory genes between bone marrow and cord tissue MSCs, especially for the “a” cluster cells (Fig. 5C). Notably, BM-High_a MSCs had higher gene expression for *PTGES2*, and the protein encoded by this gene is known to have direct or indirect role in immunomodulation by MSCs [32, 33]. *PTGES2* encodes membrane-bound prostaglandin synthase E2 which converts prostaglandin H2 (*PGH2*) to prostaglandin E2 (*PGE2*) that is known to have anti-inflammatory/immunosuppressive effects on various immune cells, including macrophages, T cells and B cells [34, 35].

MSC surface proteins are important for their significant roles in identification and functions [36]. When we compared gene expression for surface markers that are known to have some immunomodulatory functions, BM derived MSCs showed higher expression for *CD46, CD47, and CD276* whereas UCT derived MSC had higher expression for *CD81*. Surface expression of CD46 protein helps MSCs to inhibit complement binding and complement-mediated lysis [37]. *CD47* serves as a “don’t eat me” signal to avoid phagocytosis by engaging its cognate ligand signal-regulatory-protein alpha (SIRP alpha) on phagocytes [38, 39], and the interaction of *CD47* with SIRP alpha is reported to inhibit antigen presenting cell (APC) maturation and enhance STAT3 phosphorylation and IL10 induction in APC [40]. *CD276* is known to cause immune suppression by inhibiting T cell function and is currently being targeted as a check point blockade therapy for cancer [41]; however, its specific role in MSC-mediated immunomodulation is not yet confirmed. CD81 is one of the surface markers used to identify MSC-derived extracellular vehicles (EVs) but does not have any reported immunomodulatory role for MSCs; however, *CD81* coding gene is known to affect T regulatory (Treg) and myeloid-derived suppressor cell (MDSC) function, enhancing tumor growth [42]. Taken together, gene expression differences for surface markers related to immune response between BM and UCT-MSCs, implicates potential differences in the immunomodulatory functions between BM and UCT-MSCs. Further, differences in immunomodulatory gene expression between the High_a and High_b clusters for both BM and UCT-MSCs indicates functional and phenotypic heterogeneity within each MSC product. However, our in vitro functional assay, using THP-1/MSC co-culture, showed that both BM-MSCs and CT-MSCs had immunoregulatory potential. These in vitro assays fall short of predicting in vivo performance, given the complex environment encountered by the cells upon delivery. It is worth speculating that, aside from immunoregulatory potential, the ability to express genes involved in ECM organization and collagen biosynthesis may factor in cell survival within the body.

**Fig. 6:**
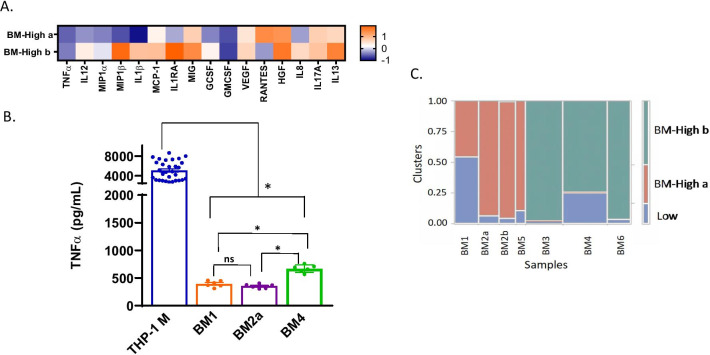
Results from Luminex are shown in a Z-score heatmap. Higher Z-score (orange) indicates positive regulation, and lower Z-score (blue) indicates negative regulation of secreted protein by MSCs. **A** Multiplex analysis of cytokines, chemokines and growth factors in the secretome of BM-MSCs in the THP-1 macrophage activation assay. **B** TNF-α suppression data for BM-MSC. One-way ANOVA with multiple comparisons (Tukey) test. THP-1 M is significant from all donors. BM1 (BM-High a cluster) is significant from BM4 (BM-High b cluster) and not from BM2a (BM-High a cluster). BM4 is significant from BM2a. These are standard error of the means. **C** Clusters of BM-MSC transcriptome profiles

**Fig. 7:**
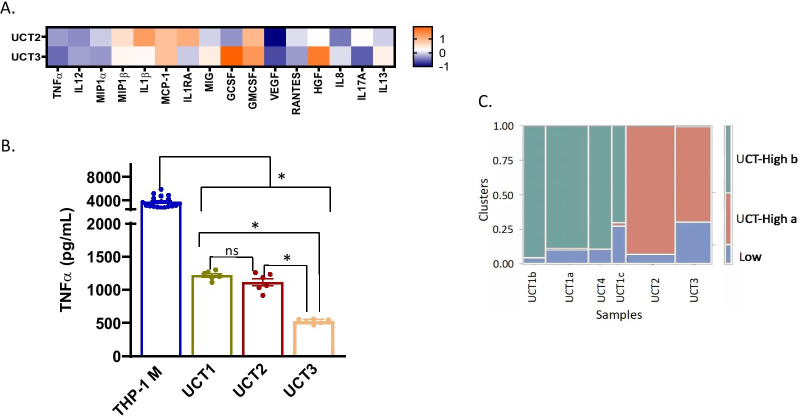
Results from Luminex are shown in a Z-score heatmap. Higher Z-score (orange) indicates positive regulation, and lower Z-score (blue) indicates negative regulation of secreted protein by MSCs. **A** Multiplex analysis of cytokines, chemokines and growth factors in secretome of UCT-MSCs in the THP-1 macrophage activation assay. **B** TNF-α suppression data for UCT-MSC. One-way ANOVA with multiple comparisons (Tukey) test. THP-1 M is significant from all donors. UCT1 (UCT-High b cluster) is not significant from UCT2 (UCT-High b cluster) but is significant from UCT3 (UCT-High b cluster). UCT2 is significant from UCT2. These are standard error of the means. **C** Clusters of UCT-MSC transcriptome profiles

We have also performed a targeted secretome assay and TNF-α Suppression assay for 3 of our BM-MSC and 3 of our CT-MSC donors. The immunomodulatory effects of BM and CT-MSCs were evaluated using a THP-1 macrophage activation assay. THP-1 monocyte-derived macrophages were stimulated with LPS and IFN-γ for 24 h then co-cultured with BM or CT-MSCs. After 24 h media were harvested and analyzed using a Human Cytokine Magnetic 30-Plex Panel for the Luminex platform (Thermo Scientific). Both BM and CT-MSCs modulated cytokine, chemokine and growth factor release and there was donor-donor variation as well as tissue-specific variation (Figs. 6, 7). We found that for out of thaw BM and CT-MSC donors were able to suppress TNF alpha (Figs. 6, 7). We see differences in the BM-MSC groups and the CT –MSCs for cytokines (IL-1β, IL-8, IL-12, and IL-17), anti-inflammatory cytokines (IL-1RA, IL-13), chemokines (MCP-1, MIG, MIP-1α, MIP-1β, and RANTES), and very subtle difference in pro-angiogenic (VEGF) markers. We see difference in growth factors (HGF and G-CSF) for the BM-MSCs and the CT-MSCs. We found significant donor differences in both UCT and BM-MSC groups which suggests our finding that cell cycle is indeed a major driving factor for the out of thaw MSC heterogeneity.

No differences in expression of a small number of pluripotent and stemness marker genes were detected between BM and UCT derived MSCs (Additional file 1: Fig. S6), though we note that abundance of these transcripts was very low which reduces power to observe differential expression.

We briefly analyzed pre-freeze and post-freeze samples from 2 donors, we identified numerous differentially expressed genes that are associated with different types of cellular functions, such as cytokine signaling, cell proliferation, cell adhesion, cholesterol/steroid biosynthesis, and regulation of apoptosis. Previously, functional differences between pre-freeze (fresh) and post-freeze MSCs were also reported by others [43, 44]. Cryopreservation has been shown to affect viability, apoptosis and metabolic activity for up to 24 h post plating and this can be tissue source or donor-related [45, 46]. These findings will need further investigation for validation.

In this study, however, we focused on in-depth scRNA-seq analysis of post-freeze MSCs as they are currently being tested as cell therapy products in many clinical trials.

In summary, this study both confirms the potential for functional differences to exist between MSCs derived from different tissues and even donors, and that within-sample heterogeneity is low. The expression of cell cycle markers is a major component of heterogeneity among donors, and manufacturing processes may need to accommodate biological and technical influences on proliferative potential. These findings will contribute to an understanding of tissue origin-based differences in therapeutic MSC products and assist in the identification of cells with immunoregulatory or persistence potential from a heterogeneous MSC population. Even though differences in the gene expression profile between bone marrow and cord tissue G0 MSC were found, further studies are needed to confirm these results, as well as the impact of these differences on the clinical use of these cells.

## Conclusions

In this study, for the first time we show 13 cryopreserved out of thaw MSC samples derived from 10 “healthy” donors, characterized at the single cell transcriptome level, which enable us with sufficient power to directly assess the contributions of donor, replicate-culture, and tissue-of-origin to gene expression, establishing that out-of-thaw bone marrow (BM)-derived and cord tissue (CT)-derived MSCs are transcriptomically highly divergent. Here, we characterized cryopreserved MSCs, immediately post-freeze, from bone marrow (BM) and cord tissue (CT), using single cell RNA sequencing (scRNAseq). We show that out-of-thaw BM- versus CT-MSCs have significant differences in gene expression. Gene-set enrichment analyses implied divergent functional potential. In addition, we show that MSC-batches can vary significantly in cell cycle status, suggesting different proliferative vs. immunomodulatory potential. Our results provide a comprehensive single-cell transcriptomic landscape of clinically and industrially relevant MSC products. We have listed potential markers found from this study, although these will need more validation and extensive study.

## Supplementary Information

**Additional file 1.** "Supplementary Information" containing figures for the pre-freeze postfreeze analysis (**Figure S1**), gene expression figures and table for key markers from BM-MSC and UCT-MSC scRNA data analysis (**Figure S3-S8, Table S1**) along with information for the statistics for the functional data (**Table S2**).

## Data Availability

The datasets used and/or analyzed during the current study are available from the corresponding author on reasonable request.

## Abbreviations

MSC: Mesenchymal stromal cell
hMSC: Human mesenchymal stromal cell
BM-MSC: Bone marrow derived MSC
UCT-MSC: Umbilical cord tissue derived MSC
scRNA-seq: Single cell RNA sequencing
