# Supplementary material for: Single-cell RNA-seq of out-of-thaw mesenchymal stromal cells shows tissue-of-origin differences and inter-donor cell-cycle variations
Source: Stem Cell Res Ther. 2021 Nov 4;12:565. doi: 10.1186/s13287-021-02627-9 (PMC8567133; doi:10.1186/s13287-021-02627-9)
Supplement: Supplementary file 1 — Additional file 1. "Supplementary Information" containing figures for the pre-freeze postfreeze analysis (Figure S1), gene expression figures and table for key markers from BM-MSC and UCT-MSC scRNA data analysis (Figure S3-S8, Table S1) along with information for the statistics for the functional data (Table S2). [file 13287_2021_2627_MOESM1_ESM.docx]

Additional file 1

Figure S1: Heatmap displaying the top 50 DE genes for the between post-thaw and pre-freeze MSC comparison. The DEG analysis between these samples shows a significant overexpression of 1,743 genes on the pre-freeze samples, compared to 310 genes significantly overexpressed in the post-thaw samples. The most significant overexpressed pathways on the Pre-freeze samples are cell proliferation and cell adhesion, while the pathways over-expressed in the frozen samples are cholesterol/Steroid biosynthesis and cell death regulation.

B.

A.

Figure S2: PCAs created with Seurat (A) Two major clusters differentiate along PC1. These clusters correspond to the low (left) and high (right) UMI count cells. (B) This PCA shows the clusters BM-High_a and BM-High_b. This cluster are not as well differentiated when using Seurat as they are when clustering the cells with SC3.

Figure S3: Dot plot displaying the MSC identity markers established by the ISCT. The size of the dots corresponds to the percentage of cells, in the tissue, expressing the gene. The color corresponds to the average non-zero expression of the gene, per cell, in each tissue. Light purple represents low expression per cell, while dark purple corresponds to high expression per cell.

Figure S4: Dot plots displaying the top 16 differentially expressed genes in each group. The first 8 genes are overexpressed in BM derived MSC while the last 8 genes are overexpressed in UCT derived MSCs.

Figure S5: Dot plot displaying the average expression for cell adhesion and migration and immunomodulatory function-associated genes. The colors represent the average expression of the genes per cell. The scale is from low expression of 0 (gray) to high expression of 1.5 log counts per million (blue). The size of the dots represents the proportion of cells in each cluster that express the gene.

A.


 B.

Figure S6: Dot plots displaying genes of interest. (A) This dot plot shows some pluripotent markers. (B) This dot plot displays stemness markers. The expression of some of these genes is low. There is not significant difference in the levels of expression between the clusters of MSCs.


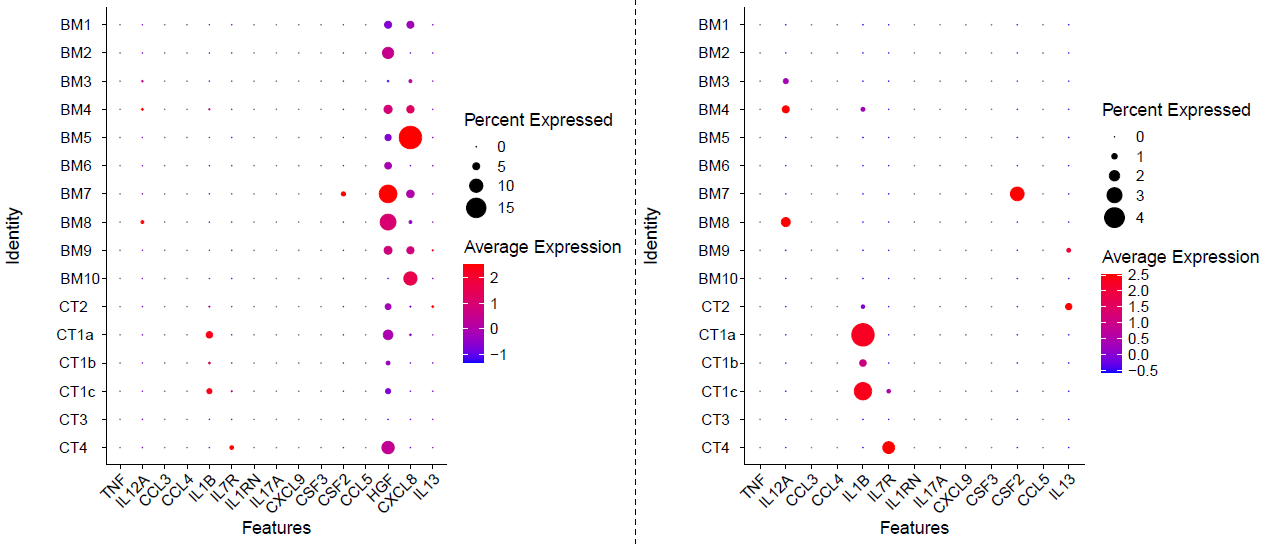


B

A

Figure S7: Dot plots displaying differences in the transcriptome level which correlates with the secretome data.


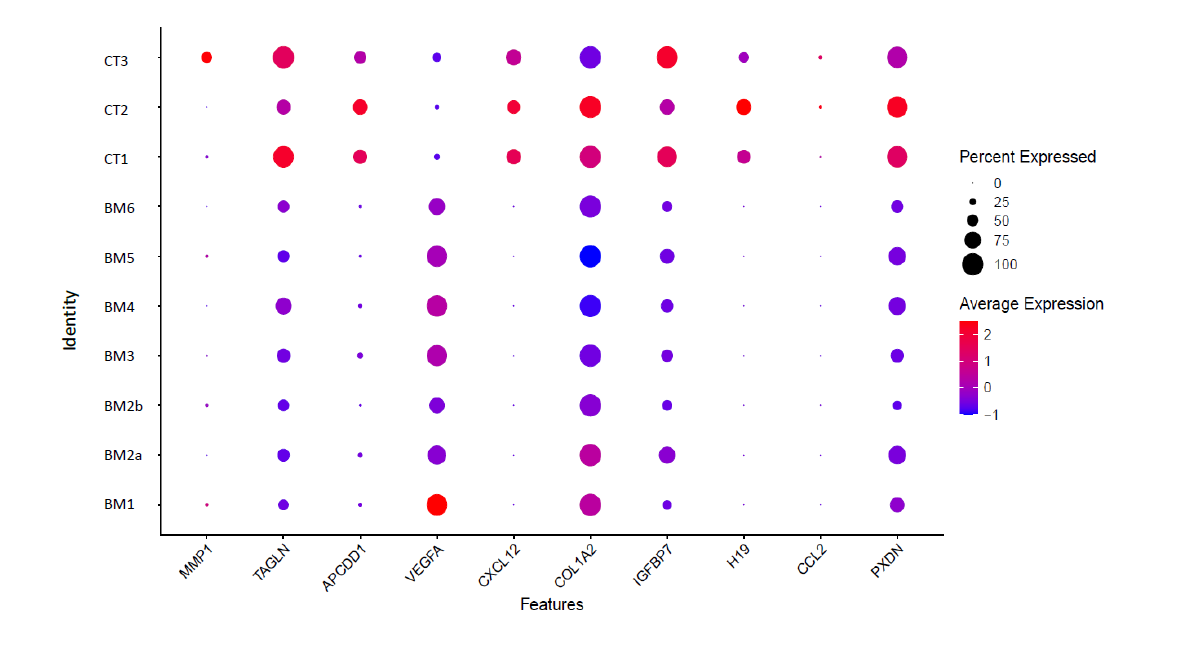


Figure S8: Dot plots displaying differences in the transcriptome level for the top gene markers differentiating BM-MSCs and CT-MSCs

| Gene ID | Function | Activated In Group |
| --- | --- | --- |
| H19 | Regulates cell differentiation | Highly expressed in CT-MSCs |
| MMP1 | Regulates MSC migration | Highly expressed in CT-MSCs |
| CXCL12 | Polarize macrophages to mitigate injury/ promotes wound repair | High expression in CT-MSCs |
| COL1A2 | Bone health regulation, used as a therapy for bone degenerative diseases | CT-MSCs shows high intensity expression of the COL1A1 gene although the BM-MSCs shows similar percentage of expression |
| PXDN | Potential stemness related marker for MSC | High expression in CT-MSCs |
| IGFBP7 | Regulates Bone metabolism. Overexpression of *IGFBP7* enhanced the expression of osteo-specific genes and proteins, and *IGFBP7* knockdown decreased osteogenesis-specific markers.  IGFBP7 knockdown in MSCs to restore proliferation and cytokine production in T-cells. These results suggest that IGFBP7 may act as a novel MSC-secreted immunomodulatory factor. | High expression is seen in CT-MSCs |
| TAGLN | MSC smooth like muscle differentiation | High expression seen in CT-MSCs |
| APCDD1 | Related to WNT pathway | High expression in CT-MSC |
| VEGFA | Promotes Bone healing | Highly expressed in BM-MSCs |

Table S1: Table discusses the potential markers here along with their functional and theorized therapeutic importance.

| Tukey's multiple comparisons test | Mean Diff. | 95.00% CI of diff. | Below threshold? | Summary | Adjusted P Value |
| --- | --- | --- | --- | --- | --- |
| UCT1 vs. UCT2 | 104.1 | -25.75 to 234.0 | No | ns | 0.1754 |
| UCT1 vs. UCT3 | 695.1 | 565.2 to 825.0 | Yes | **** | <0.0001 |
| UCT1 vs. BM1 | 825.9 | 696.0 to 955.7 | Yes | **** | <0.0001 |
| UCT1 vs. BM2a | 868.2 | 738.3 to 998.1 | Yes | **** | <0.0001 |
| UCT1 vs. BM4 | 550.1 | 420.3 to 680.0 | Yes | **** | <0.0001 |
| UCT2 vs. UCT3 | 590.9 | 461.1 to 720.8 | Yes | **** | <0.0001 |
| UCT2 vs. BM1 | 721.7 | 591.8 to 851.6 | Yes | **** | <0.0001 |
| UCT2 vs. BM2a | 764.1 | 634.2 to 893.9 | Yes | **** | <0.0001 |
| UCT2 vs. BM4 | 446.0 | 316.1 to 575.9 | Yes | **** | <0.0001 |
| UCT3 vs. BM1 | 130.8 | 0.8828 to 260.7 | Yes | * | 0.0477 |
| UCT3 vs. BM2a | 173.1 | 43.22 to 303.0 | Yes | ** | 0.0041 |
| UCT3 vs. BM4 | -144.9 | -274.8 to -15.06 | Yes | * | 0.0218 |
| BM1 vs. BM2a | 42.33 | -87.55 to 172.2 | No | ns | 0.9171 |
| BM1 vs. BM4 | -275.7 | -405.6 to -145.8 | Yes | **** | <0.0001 |
| BM2a vs. BM4 | -318.0 | -447.9 to -188.2 | Yes | **** | <0.0001 |

Table S2: Comparing all UCT and BM groups with ANOVA and Tukey post hoc comparisons for the secretome assay.
